# A Walk through Recent Nitro Chemistry Advances

**DOI:** 10.3390/molecules25163680

**Published:** 2020-08-12

**Authors:** Nagatoshi Nishiwaki

**Affiliations:** Research Center for Molecular Design, Kochi University of Technology, Tosayamada, Kami, Kochi 782-8502, Japan; nishiwaki.nagatoshi@kochi-tech.ac.jp; Tel.: +81-887-57-2517

**Keywords:** nitro group, conjugate addition, 1,3-Dipole, electron-withdrawing ability, electrophilicity, nitration, nitronate, nucleophilicity

## Abstract

Chemistry of nitro groups and nitro compounds has long been intensively studied. Despite their long history, new reactions and methodologies are still being found today. This is due to the diverse reactivity of the nitro group. The importance of nitro chemistry will continue to increase in the future in terms of elaborate synthesis. In this article, we will take a walk through the recent advances in nitro chemistry that have been made in past decades.

## 1. Introduction

The chemistry of nitro compounds began at the beginning of the 19th century and has developed together with organic chemistry; in the 20th century, various reactivity properties of nitro groups were elucidated. Nitro compounds play an important role as building blocks and synthetic intermediates for the construction of scaffolds for drugs, agricultural chemicals, dyes, and explosives. In the world, millions of tons of nitro compounds are synthesized and consumed every year. In the 21st century, researchers’ attentions gradually shifted to the use of nitro compounds in the elaborate syntheses such as controlling reactivity and stereochemistry. Development of new synthetic methods has also progressed using a combination of the diverse properties of nitro groups in past decades. Indeed, numerous methodologies are reported in current scientific journals. In this article, I would like to touch lightly on the recent advances in the chemistry of nitro compounds. For more information, please see the review articles cited in the references.

## 2. Nitration

Nitration is one of the fundamental chemical conversions. Conventional nitration processes involve HNO_3_ alone or in combination with H_2_SO_4_, and this method has remained unchallenged for more than 150 years. Although other nitrating agents have been employed in a laboratory, these are not applicable to large-scale reactions because harsh conditions are sometimes necessary. The conventional methods also suffer from large amounts of waste acids and difficulty of regiocontrol [1,2]. These problems are overcome by using solid acids such as zeolites. High *para*-selective nitration was achieved by using tridirectional zeolites H_β_ [3] because of the steric restriction when substrate is adsorbed in the zeolite cavity [4].

Suzuki et al. developed an excellent nitration method using NO_2_ and O_3_, referred to as the Kyodai method [5]. This reaction proceeds efficiently even at low temperature. The addition of a small amount of a proton acid or Lewis acid enhances reactivity of the substrate to enable the polynitration.

Since nitrating agents also serve as strong oxidants, nitro compounds are often accompanied by oxidation products [6]. In order to avoid the formation of byproducts and regioisomers, *ipso*-nitration methods have been developed. Wu et al. showed metal-free nitration using phenylboronic acid and *t*-BuONO to afford nitrobenzene [7]. Furthermore, Buchwald et al. reported palladium catalyzed *ipso*-nitration method using chlorobenzene and commercially available NaNO_2_ [8].

With the recent development of research on transition metal-catalyzed C-H activation, various skeletons have been constructed. Nitroaromatic compounds are obtained by this protocol, in which the directing group facilitates the regioselective nitration [9].

## 3. Reactivity and Application

The versatile reactivity of the nitro compounds family originates from the diverse properties of the nitro group. The strong electron-withdrawing nitro group reduces the electron density of the scaffold framework through both inductive and resonance effects, which undergoes reactions with nucleophiles or single-electron transfer. Makosza et al. indicated that reactions of nitroarenes with nucleophiles proceed through either direct nucleophilic attack forming σ-adduct or single-electron transfer forming a radical-ion pair [10,11,12].

The α-hydrogen is highly activated by the adjacent strong electron-withdrawing ability of the nitro group, which facilitates the α-arylation upon treatment of nitroalkanes with various arylating reagents leading to pharmaceutically active molecules [13]. The α-hydrogen is also acidic to attract basic reagents that are close together, and the spatial proximity undergoes an efficient reaction— similar to an intramolecular process—to afford polyfunctionalized compounds, which are referred to as a pseudo-intramolecular process [14]. The acidic hydrogen accelerates the tautomerism between nitroalkane and nitronic acid, among which the latter reveals high electrophilicity to react with carbon nucleophiles [15].

The nitro group stabilizes α-anion (nitronate ion), which serves as a nucleophile. Recently, the stereoselective Henry reaction (with aldehydes) [16,17] and nitro-Mannich reaction (with imine) [18] have been established, leading to enantiomerically rich β-nitroalcohols and β-nitroamines, respectively. Recent advances are noteworthy for the asymmetric organocatalytic conjugate addition of nitroalkanes to α,β-unsaturated carbonyl compounds [19,20]. Nitro group activates the connected carbon–carbon double bond, which serves as an excellent Michael acceptor to construct versatile frameworks [21,22,23]. These reactivities reveal significant utility in elaborate syntheses. Indeed, a lot of natural products have been synthesized using stereoselective reactions [24].

Nitro group also activates the connected carbon–carbon triple bond, however, it is too reactive to be used practically. The first synthesis of nitroalkyne was achieved in 1969 by Viehe [25]. During the subsequent half century, development of the synthetic methods and studies on reactivity, as well as physical/chemical properties, has progressed [26].

Deprotonated nitroalkane (nitronate) is characterized by the dual nature of nucleophilic and electrophilic properties. Indeed, versatile reactivities are used for synthesizing complex frameworks [27,28]. The dual nature of the nitronate also facilitates the 1,3-dipolar cycloadditions leading to functionalized heterocyclic compounds, which are not readily available by an alternative method [29,30].

Besides activating ability for the scaffold, the nitro group also serves as a good leaving group in organic reactions. A carbon–carbon double bond is formed upon the elimination of a HNO_2_ from nitroalkane, which was energetically studied by Ballini et al. [12,31]. The combination of roles as an activator and as a leaving group enables the synthesis of polyfunctionalized compounds [32,33]. Furthermore, nitrobenzenes can be used as substrates for the transition-metal catalyzed cross-coupling, in which the nitro group is substituted with various nucleophiles [34].

Moreover, synthetic utility of the nitro group is improved by adding the chemical conversion to the abovementioned properties. The most fundamental transformation of the nitro group is reduction, which converts a nitro group to nitroso, oxime and amino groups. Vast numbers of combinations of catalysts and reducing agents have been developed for this purpose. Especially, recent progress of reduction using metal nanoparticles is noteworthy [35,36,37]. The landmark of the functional group conversion is the Nef reaction, which transforms a nitroalkane to the corresponding ketone. Since the first report at the end of 19th century [38], the usefulness of this reaction has not diminished, and it is still widely used in organic syntheses [39]. The chemical diversity of a nitro group enables us to construct a compound library possessing versatile electronic structure, which is helpful for developing new functional materials such as dyes and optical/electronic materials [40].

Due to the unique chemical behavior (reactivity and functional group conversions), nitro compounds serve as the synthetic intermediates for various types of compounds. In addition, nitro compounds themselves reveal specific properties. The explosive materials have been used in various situations such as the construction industry, mining minerals, processing metals and synthesis of nanomaterials, in which nitro compounds have played an important role [41]. Recent progress in this area provided more powerful explosive nitro compounds containing plural nitrogen. Although nitro compounds seem to be common in artificial materials, natural products containing a nitro group have been isolated from plants, fungi, bacteria, and mammals [42]. Accordingly, they exhibit biological activity. Indeed, many drugs containing a nitro group have been developed [43,44].

## 4. Conclusions

Chemistry of the nitro group and nitro compounds has been energetically investigated for a long time. Despite the long history including numerous reports, new reactions and methodologies are found even now. The unique physical/chemical properties of the nitro group will facilitate the progress of organic/inorganic chemistry and material science. Hence, nitro chemistry will continue to be increasingly important in the future.
